# Pharmacokinetic Study of Praziquantel Enantiomers and Its Main Metabolite *R-trans*-4-OH-PZQ in Plasma, Blood and Dried Blood Spots in *Opisthorchis viverrini*-Infected Patients

**DOI:** 10.1371/journal.pntd.0004700

**Published:** 2016-05-06

**Authors:** Isabel Meister, Jana Kovac, Urs Duthaler, Peter Odermatt, Jörg Huwyler, Fiona Vanobberghen, Somphou Sayasone, Jennifer Keiser

**Affiliations:** 1 Department of Medical Parasitology and Infection Biology, Swiss Tropical and Public Health Institute, Basel, Switzerland; 2 University of Basel, Basel, Switzerland; 3 Department of Epidemiology and Public Health, Swiss Tropical and Public Health Institute, Basel, Switzerland; 4 Department of Pharmaceutical Sciences, Division of Pharmaceutical Technology, University of Basel, Basel, Switzerland; 5 National Institute of Public Health, Ministry of Health, Vientiane, Lao People’s Democratic Republic; McGill University, CANADA

## Abstract

**Background:**

Praziquantel (PZQ) is the treatment of choice for infections with the liver fluke *Opisthorchis viverrini*, a major health problem in Southeast Asia. However, pharmacokinetic (PK) studies investigating the disposition of PZQ enantiomers (*R*- and *S*-PZQ) and its main metabolite, *R-trans*-4-OH-PZQ, in diseased patients are lacking. The implementation of a dried blood spot (DBS) sampling technique would ease the performance of PK studies in remote areas without clinical facilities. The aim of the present study is to provide data on the disposition of PZQ enantiomers and *R-trans*-4-OH-PZQ in opisthorchiasis patients and to validate the use of DBS compared to plasma and blood sampling.

**Methodology/Principal Findings:**

PZQ was administered to nine *O*. *viverrini*-infected patients at 3 oral doses of 25 mg/kg in 4 h intervals. Plasma, blood and DBS were simultaneously collected at selected time points from 0 to 24 h post-treatment. PK parameters were determined using non-compartmental analysis. Drug concentrations and areas under the curve (AUC_0–24h_) measured in the 3 matrices were compared using Bland-Altman analysis. We observed plasma AUC_0–24h_s of 1.1, 9.0 and 188.7 μg/ml*h and half-lives of 1.1, 3.3 and 6.4 h for *R*-PZQ, *S*-PZQ and *R-trans*-4-OH, respectively. Maximal plasma concentrations (C_max_) of 0.2, 0.9 and 13.9 μg/ml for *R*-PZQ, *S*-PQZ and *R-trans*-4-OH peaked at 7 h for PZQ enantiomers and at 8.7 h for the metabolite. Individual drug concentration measurements and patient AUC_0–24h_s displayed ratios of blood or DBS *versus* plasma between 79–94% for *R*- and *S*-PZQ, and between 108–122% for *R-trans*-4-OH.

**Conclusions/Significance:**

Pharmacodynamic (PD) in vitro studies on PZQ enantiomers and *R-trans*-4-OH-PZQ are necessary to be able to correlate PK parameters with efficacy. DBS appears to be a valid alternative to conventional venous sampling for PK studies in PZQ-treated patients.

## Author Summary

Opisthorchiasis, caused by the food-borne trematode *Opisthorchis viverrini*, affects more than 8 million people in Southeast Asia, and in its chronic phase it might lead to cholangiocarcinoma. Praziquantel (PZQ) is the sole drug available to treat the disease and is administered as a racemic mixture of *R* and *S* enantiomers, of which *R*-PZQ is considered active. As PZQ is rapidly metabolized, its disposition and efficacy in patients might considerably vary according to disease state, sex or age. However, pharmacokinetic (PK) studies on the disposition of PZQ enantiomers and its main metabolite, *R-trans*-4-OH, in diseased patients are lacking. To allow the collection of PK samples in a large number of patients, we implemented a dried blood spot (DBS) technique, which is less invasive than venipuncture. The aim of our study is to provide first data on the disposition of PZQ enantiomers and the main metabolite of PZQ in opisthorchiasis patients and to validate the use of DBS over venous sampling. Standard PZQ treatment was administered to nine *O*. *viverrini* infected patients, and plasma, blood and DBS were simultaneously collected within 24 h post-treatment. We observed a 100-fold higher disposition of the metabolite compared to *R*-PZQ, which questions its role in the opisthorchidal activity of PZQ. DBS sampling appears to be a valid alternative to venous sampling and will be a valuable tool for future PK studies in PZQ-treated patients.

## Introduction

Opisthorchiasis is caused by the trematode *Opisthorchis viverrini*, a liver fluke affecting about 8 million people in Southeast Asia, particularly in the Mekong basin [1, 2]. Infection occurs following consumption of raw or undercooked fish harboring *O*. *viverrini* metacercariae [3]. In the early phase, the disease is mostly asymptomatic but in the acute stage periductal fibrosis and liver enlargement are common, mostly a result of inflammation due to worm feeding. The chronic stage triggers severe clinical symptoms including jaundice, biliary obstructions, and cholangiocarcinoma as a serious complication [4–7].

Praziquantel (PZQ) is the drug of choice for opisthorchiasis and is manufactured as a racemic mixture of *R* and *S* enantiomers. The recommended treatment regimen is 3 oral doses of 25 mg/kg, usually administered between 4 and 6 h apart [8]. The disposition of PZQ is highly influenced by the fasting state, the co-administered food type, as well as the liver function [9, 10]. Though no studies have been conducted against *O*. *viverrini* yet, *R*-PZQ is considered to be the active molecule in the treatment of schistosomiasis, while the inactive *S*-PZQ is suspected to be responsible for the bitter taste of the drug and for the mild to moderate adverse events caused by the treatment [11–14]. In humans, PZQ undergoes an enantioselective first-pass metabolism through the cytochrome CYP450 3A4 isoform [15] and is mainly transformed into the monohydroxylated metabolite *R-trans*-4-OH-PZQ (*R-trans*-4-OH), while *S-*PZQ is metabolized to several different monohydroxylated molecules [16–18]. *R-trans*-4-OH displays minor anthelmintic activity, with an IC_50_ hundred times higher than *R*-PZQ against *Schistosoma mansoni* [19].

The disposition of PZQ enantiomers and metabolites has not yet been studied in opisthorchiasis patients. In fact, the only pharmacokinetic (PK) study of PZQ involving patients with opisthorchiasis focuses on the racemic drug [20]. Enantioselective disposition was performed exclusively in healthy volunteers with a low dose [21]. Therefore, studies on the enantioselective disposition of PZQ in the diseased population are warranted for a better understanding of the modalities of drug action and disposition.

Dried blood spot (DBS) sampling is a microsampling technique, involving the collection of capillary blood through a finger prick. The method is therefore less invasive compared to venipuncture. The blood drops are dried on a filter paper and stored at ambient temperature until assayed. Compared to blood or plasma sampling, this method does not require sample freezing and offers easy handling and storage, hence allowing the performance of PK studies in remote areas without clinical set-ups. Blood quantities withdrawn with DBS are minimal (20 μl *vs*. 3–4 ml for plasma or blood), which provides an advantage for research with children. Finally, the ease of sample collection enables including a larger number of patients and is therefore ideal for population PK studies [22–25]. The major caveat when replacing plasma with DBS sampling is the use of a different matrix where the drug partition might not be equivalent [25, 26]. Validating this alternative sampling technique for future trials hence calls for a formal comparison of drug concentrations measured in plasma and DBS.

The aim of our study was to elucidate for the first time the kinetic disposition of both PZQ enantiomers and its main metabolite in *O*. *viverrini*-infected patients. Additionally, we assessed the difference between concentrations determined in plasma, blood and DBS sampling for the analysis of PZQ PK profiles using Bland-Altman analysis.

## Materials and Methods

### Chemicals and reagents

**Fig 1:**
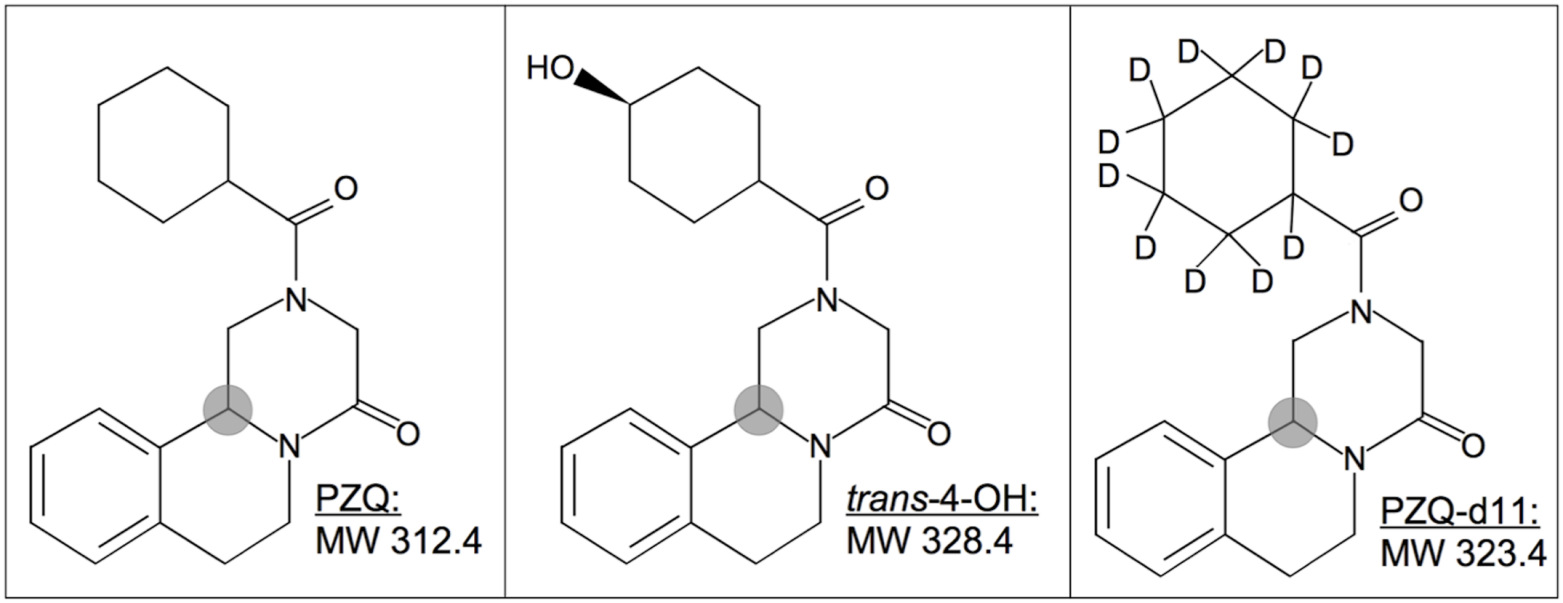
Chemical structures of PZQ, the main metabolite *trans*-4-OH and PZQ-d11 (internal standard), with the chiral centre represented with a shaded circle.

Racemic (rac) PZQ was obtained from Sigma-Aldrich (Buchs, Switzerland). PZQ enantiomers as well as the metabolite *trans*-4-OH were donated by Merck Serono (Darmstadt, Germany). Eleven-fold deuterized PZQ (PZQd11, internal standard-IS) was acquired from Toronto Research Chemicals (Ontario, Canada). The chemical structures of PZQ, PZQd11 and *trans*-4-OH are depicted in Fig 1. Acetonitrile, ethanol and methanol of MS grade were purchased from Carl Roth GmBH (Allschwil, Switzerland), and ammonium formate, ammonium acetate and formic acid of MS grade from Sigma-Aldrich (Buchs, Switzerland). Ultrapure water was provided using a Millipore Milli-Q water purification system (Merck Millipore, Darmstadt, Germany). Blank human plasma and blood were supplied in lithium heparin-coated vacutainer tubes (BD, Allschwil, Switzerland) from the local blood donation centre (Basel, Switzerland).

### PK sample collection

The plasma, blood and DBS sample collection was performed in the framework of a PK and dose-finding study of tribendimidine against *O*. *viverrini* in humans. Ethical clearance was obtained from the ethics committee of Northern and Central Switzerland (EKNZ reference no. 375/11), and from the National Ethic Committee for Health Research, Ministry of Health (MoH) of Lao PDR (reference no. 009/NECHR). The trial is registered at Current Controlled Trials (ISRCTN96948551). In short, 9 *O*. *viverrini*-infected patients were treated with 3 oral doses of 25 mg/kg PZQ, with the second and third dose administered 4 and 8 h after the first dose, respectively. The trial was performed at the Champasak Provincial Hospital in Pakse, Lao PDR, and prior to treatment, a standardized food dish (rice) was provided to all patients. Adverse events were monitored at 3, 24 and 48 hours post-treatment using a standardized questionnaire. Prior to treatment, patients underwent physical examinations and laboratory tests, such as liver and kidney parameters and complete blood counts. About 4 ml of venous blood was collected at 0, 2, 4, 6, 8, 8.5, 9, 10, 11, 12 and 24 h after the first dose from the antecubital arm vein through an intravenous catheter into EDTA-coated vacutainer tubes (BD). Within 30 min after sampling, 1 ml of blood was pipetted into a cryotube and the remaining blood centrifuged to obtain plasma. Plasma and blood samples were transported on dry ice to Basel where they were kept at -80°C until analysis. DBS samples were collected at 0, 4, 8, 9, 11 and 24 h post-first-dose from patients 1 to 5, and at 0, 2, 6, 8.5, 10 and 12 h post-first-dose from patients 6 to 9. The samples were obtained by puncturing the middle or ring finger with sterile one-way finger prickers (Accu-Check Safe-T-Pro Plus, Roche, Switzerland). Lithium heparin coated capillaries (Alere Cholestech LDX, V = 40 μl) were used to collect and deposit blood on DMPK-C cards (Whatman, GE Healthcare Life Sciences, Cardiff, UK). The cards were dried overnight and stored in plastic bags with desiccant at room temperature.

### Analytical method

The LC-MS/MS method for the analysis of *R*- and *S*-PZQ and *R-trans*-4-OH and its validation for plasma, blood and DBS is described elsewhere [27]. Briefly, plasma and blood calibration samples were freshly prepared and included in each analytical run by spiking blank samples to reach final concentrations from 2.5 to 0.01 (lower limit of quantification-LLOQ) μg/ml for *R*- and *S*-PZQ, and of 25 to 0.1 (LLOQ) μg/ml for *R-trans*-4-OH. QC samples were similarly prepared by spiking 6 different blanks to obtain high, medium, low and LLOQ concentrations. For the extraction of analytes, 100 μl of plasma or blood samples underwent protein precipitation with 700 μl of IS solution (500 ng/ml IS in pure acetonitrile), and were shaken in a thermomixer for 20 min at 25°C and 1400 rpm. DBS samples of 5 mm diameter were extracted with 300 μl of DBS extraction solution (IS solution: ultrapure water, 4:1, v/v), shaken in a thermomixer for 20 min at 25°C, and sonicated for 40 min prior analysis.

A first chromatographic separation was achieved through a column trapping system (HALO C-18, 4.6 x 5 mm, Optimize Technologies, OR, USA) using 10 mM ammonium acetate and 0.15% formic acid in ultrapure water at a flow rate of 0.3 ml/min. After 1 min, the analytes were eluted from the trapping to the chiral column (Lux Cellulose-2 (150x4.6mm, 3μm, Phenomenex, CA, USA)) with an elution gradient of 70 to 90% B, with mobile phase A consisting of 20 mM ammonium formate in ultrapure water and mobile phase B of pure acetonitrile.

### Treatment efficacy and pharmacokinetic analysis

Statistical analyses were performed with Prism software (GraphPad, CA, USA). Parasite egg counts were determined with duplicate Kato-Katz smears from two stool samples prior to the treatment and between 19 and 25 days after treatment for the estimation of treatment efficacy. Cure rates were defined as the percentage patients who were egg-negative after treatment. The number of eggs per gram of stool (EPG) was evaluated by adding up the egg counts from the quadruplicate Kato-Katz thick smears and multiplying this number by a factor of six. Geometric mean egg counts were calculated before and after treatment to determine the corresponding percentage egg-reduction rate (ERR).

To evaluate the reproducibility of the measurements, incurred sample reanalysis (ISR) was performed with a total of 170 samples originating from 5 patients in the 3 matrices (56% of total sample size). The percentage difference between the original and the reanalyzed measurement was calculated as follows:

$$percentage\;difference=\frac{(repeat-original)\times 100}{mean\;(repeat,\;original)}$$

As acceptance criterion for ISR, at least 66.7% of the samples (2 out of 3) should not deviate by more than 20%, as recommended in the European guidelines on bioanalytical method validation and the daft of the FDA guidelines [28, 29].

PK parameters, including the area under the concentration-time curve (AUC_0–24h_), the maximal concentration (C_max_), the time to maximal concentration (T_max_) and the half-life (t_1/2_) were calculated for each patient with the Excel add-in PKsolver [30] using non-compartmental analysis with the linear trapezoidal rule.

### Agreement between matrices

Concordance of drug concentrations observed in blood or DBS compared to plasma was evaluated using Pearson’s correlation coefficient and Bland-Altman plots, with percentage ratios between the two matrices (blood/plasma or DBS/plasma) plotted against mean concentrations [31–33]. For matrix differences in AUC values, Bland-Altman analysis also applied.

The limits of agreement at 95% for the ratios were calculated as follows:

limits of agreement=mean percentage ratio ± 1.96× standard deviation

For the drug concentration data, the calculation of the limits of agreement were adapted to take into account multiple measurements per individuals, following the method described by Bland and Altman [34] using Stata software (version 12.1, College State, TX, USA).

### *In vitro* assessment of blood/plasma partition of PZQ

The partitioning of PZQ between plasma and erythrocytes was assessed *in vitro* using human blood from the local blood donation center. Whole blood samples (hematocrit adjusted to 35%) were spiked in triplicate with the analytes of interest to reach end concentrations of 0.05 and 0.5 μg/ml for *R*- and *S*-PZQ and 0.5 and 5 μg/ml for *R-trans*-4-OH. The samples were incubated 1 h at room temperature. Hundred microliters of each sample were aliquoted and the remainder was centrifuged at 1500 g for 20 minutes to obtain plasma. Whole blood and plasma samples were extracted using acetonitrile containing IS and analyzed as described above. Analyte peaks were normalized with IS peaks and ratios of blood to plasma were calculated for each concentration and analyte.

## Results

### Sample collection and analysis

A total of 91 plasma and 91 blood samples were collected. For DBS, 45 samples were analysed. Due to technical problems, five DBS samples for patient 9 were collected at 6, 7, 8.5, 10 and 12 h post-treatment, and an extra venous blood sample was withdrawn at 7 h post-treatment. To estimate the repeatability of the analytical measurements, an incurred sample reanalysis was performed. Between 88.2 and 100% of the samples in plasma, blood and DBS were within the ISR acceptance criterion (within 20% difference). All samples from the 9 patients presenting obvious measurement errors or displaying a high discrepancy between matrices were reanalysed (n = 21).

For *R*-PZQ, concentrations ranged from 0.01 to 0.85 μg/ml, to 0.90 μg/ml and to 1.08 μg/ml for DBS, blood and plasma, respectively. For *S*-PZQ, the following concentration ranges were observed: 0.01–1.59 μg/ml in DBS, 0.01–1.83 μg/ml in blood, and 0.02–2.34 μg/ml in plasma. The metabolite *R-trans*-4-OH displayed concentrations ranging from 3.01 to 19.01 μg/ml in DBS, 1.62 to 22.05 μg/ml in blood, and 1.41 to 17.85 μg/ml in plasma.

### Treatment efficacy and kinetic disposition of PZQ in patients

All participants were adults, 3 males and 6 females aged 25 to 46 years with a median weight of 56 kg (Table 1). Prior to treatment, 8 patients displayed moderate *O*. *viverrini* infections (between 1,000 and 10,000 EPG) and 1 patient a heavy infection (13,920 EPG). All patients were asymptomatic. Hookworm co-infections were present in 5 participants (participants 1, 4, 6, 7 and 8), while patient 2 presented a co-infection with the whipworm *Trichuris trichiura*. Liver and kidney parameters were in the normal range for all the patients. Blood counts were also normal, except for patient 2 who displayed slightly elevated white blood cell counts (11.7 * 10^3^ cells/l) and a moderate anaemia (hemoglobin concentration = 9.2 g/dl). All patients were treated as planned and tolerated the treatment well, with the exception of patient 2, for whom the treatment was interrupted due to adverse events (vomited within 30 min after the second dose). Between 19 and 25 days post-treatment, the participants were screened again for the presence of *O*. *viverrini* eggs in stool: all patients were cleared from infection, hence cured (Table 1).

**Table 1:** Characteristics of participants and *O*. *viverrini* cure rates.

Median age (range) [years]	Sex [% females]	Median weight (range) [kg]	Pre-treatment [EPG] Geometric mean	Post-treatment [EPG] Geometric mean	Cure rate [%]
40 (25–46)	67	56 (40–77)	3653	0	100

**Fig 2:**
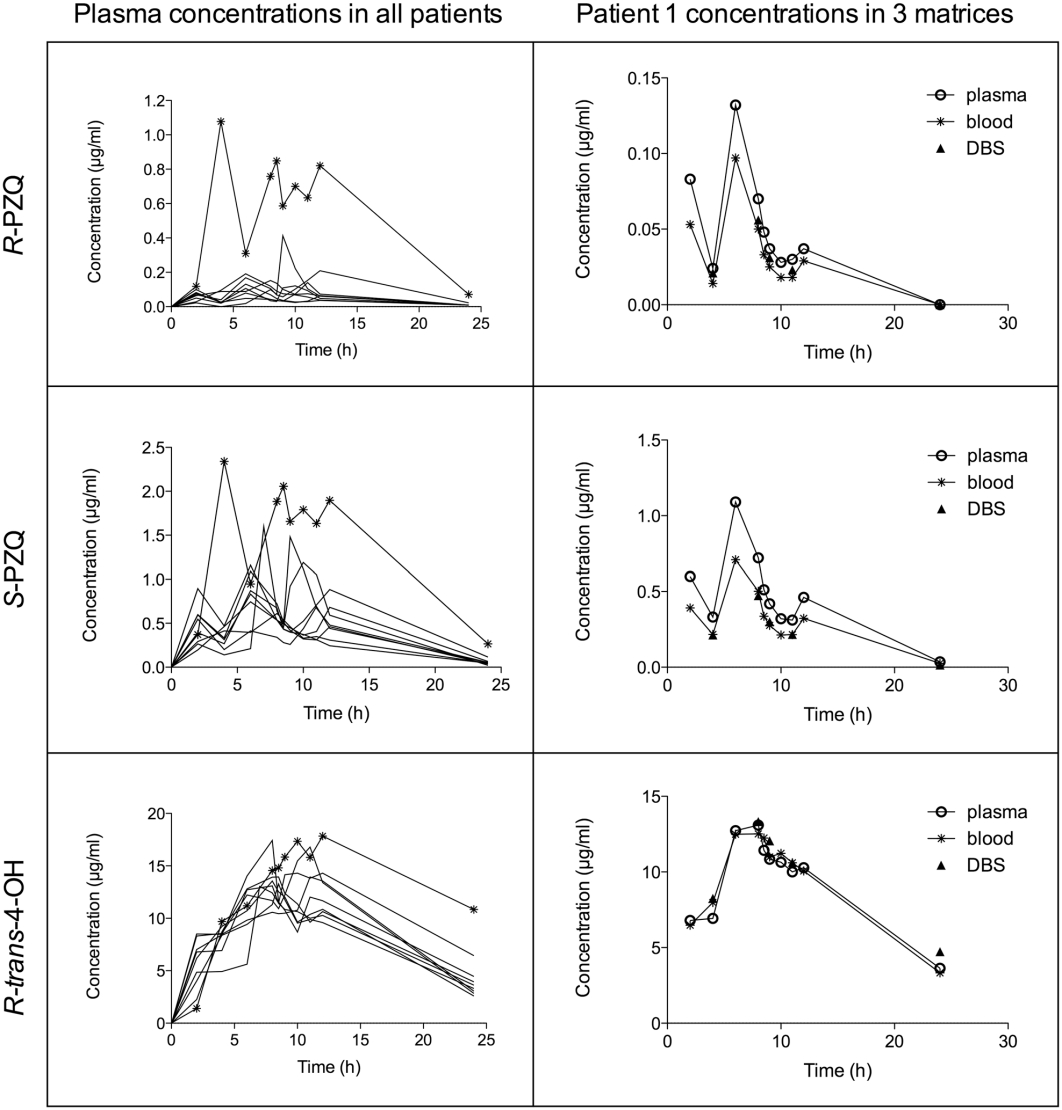
Plasma concentrations for *R*-PZQ (1), *S*-PZQ (2) and *R-trans*-4-OH (3) over time and across patients (patient 2 sketched with a star) and analyte concentrations across the 3 matrices in patient 1.

Patient variability in plasma concentrations was high, with patient 2 displaying clearly higher concentrations than the other subjects, despite not taking the last dose (Fig 2). Median PK parameters calculated from plasma concentrations are summarized in Table 2. *R*-PZQ displays the smallest AUC_0–24h_ (1.1 μg/ml*h) and a short estimated t_1/2_ (1.1 h) compared to the other analytes. *S*-PZQ exhibits a nearly 5 x higher C_max_ (0.9 μg/ml) and an AUC_0–24h_ more than 8 x higher than *R*-PZQ (9.0 μg/ml*h). Both enantiomers peak at the same time (7 h). The main metabolite *R-trans*-4-OH has an increased exposure compared to the parent molecule. For example, its AUC_0–24h_ (188.7 μg/ml*h) is 20x greater than *S*-PZQ and 170x greater than *R*-PZQ. The metabolite’s estimated t_1/2_ and T_max_ are 6.4 h and 8.7 h, respectively. Patient 2 displays 2–10 fold higher *R*-PZQ, *S*-PZQ and *R-trans*-4-OH AUC_0–24h_ values compared to the other patients.

**Table 2:** Median (range) of AUC_0–24h_, t_1/2_, C_max_ and T_max_ in plasma across 9 patients.

Analyte	AUC_0–24h_ [μg/ml*h]	t_1/2_ [h]	C_max_ [μg/ml]	T_max_ [h]
*R*-PZQ	1.1 (0.8–10.7)	1.1 (1.0–3.0)	0.2 (0.1–1.1)	7.00 (4.0–11.8)
*S*-PZQ	9.0 (6.1–26.3)	3.3 (1.9–3.7)	0.9 (0.6–2.3)	7.00 (4.0–11.8)
*R-trans*-4-OH	188.7 (157.2–257.4)	6.4 (4.1–7.1)	13.9 (13.1–17.9)	8.7 (8.0–12.0)

### Sample measurement agreement between matrices

When comparing the analyte concentrations obtained in the different matrices by Pearson’s correlation coefficient, blood *versus* plasma and DBS *versus* plasma data displayed correlation coefficients above 0.92 (all p <0.01, Table 3). The mean concentration curves are consistent for plasma, blood and DBS, as exemplified with patient 1 (Fig 2).

**Table 3:** Concordance of blood and DBS compared to plasma measurements, evaluated with Pearson’s correlation and percentage ratio with their 95% limits of agreement (LoA).

Analyte	Matrix	Correlation coefficient	Percentage ratio^a^ (LoA^b^) [%]
*R*-PZQ	blood (n = 91)	0.995	79.0 (59.1; 99.0)
	DBS (n = 45)	0.994	89.6 (55.6; 123.6)
*S*-PZQ	blood (n = 91)	0.963	93.9 (54.5; 133.3)
	DBS (n = 45)	0.970	92.1 (44.4; 139.8)
*R-trans*-4-OH	blood (n = 91)	0.948	122.0 (94.0; 145.0)
	DBS (n = 45)	0.921	110.6 (77.0; 144.3)

^a^The percentage ratio is computed as blood or DBS values divided by plasma measurements averaged across patients and time points and presented as percentage to the plasma values

^b^The 95% limits of agreement for the individual data were calculated with the modified Bland-Altman method for multiple measurements per individual

**Fig 3:**
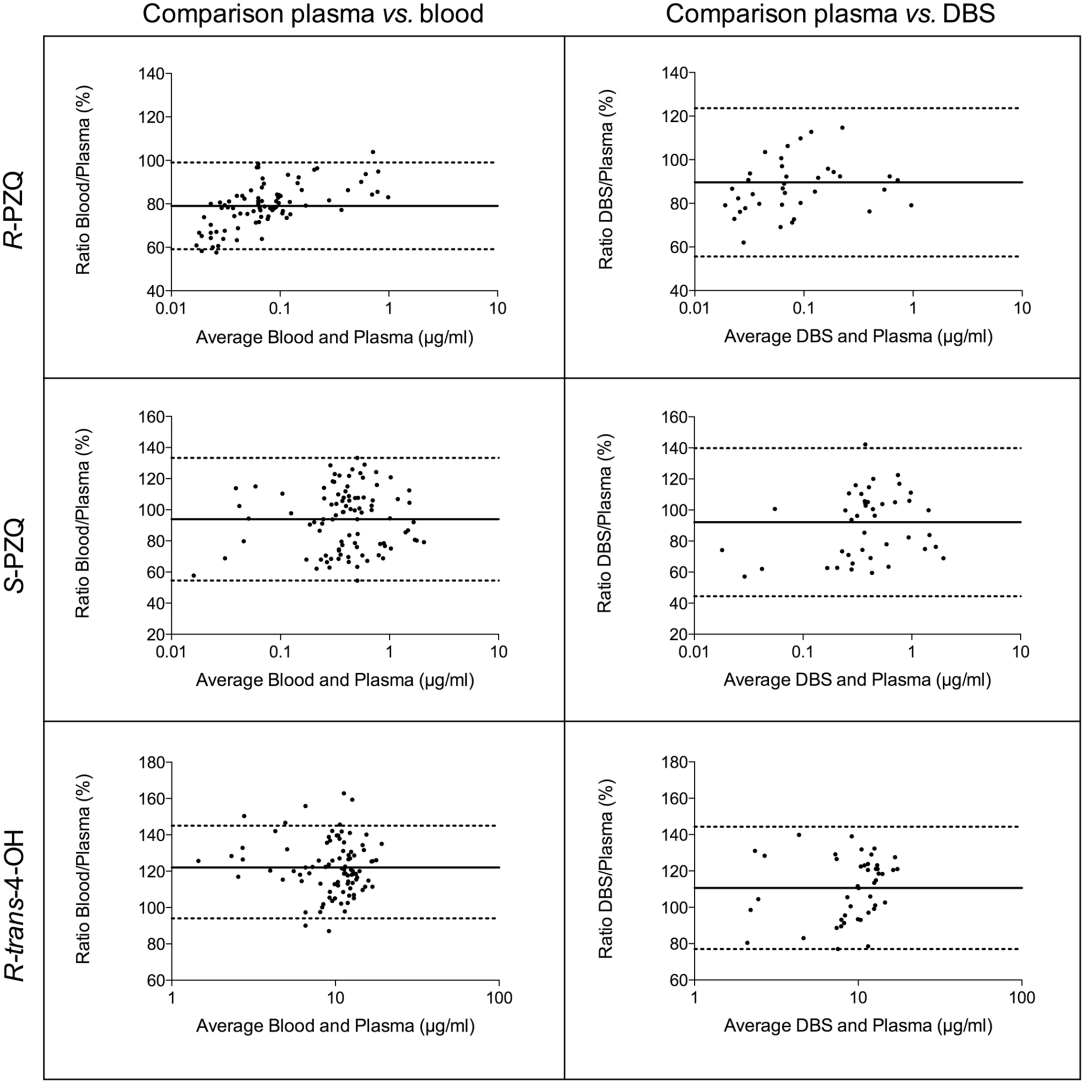
Bland-Altman plots of measurement performed with blood or DBS compared to plasma values for *R*-PZQ, *S*-PZQ and *R-trans*-4-OH with mean ratio sketched with a solid line and 95% limits of agreement with dashed lines.

The modified Bland-Altman approach for multiple measurements per individual was used on drug concentrations, although the values obtained with this method did not differ from the conventional approach. The Bland-Altman plots (Fig 3) show that percentage ratios were generally consistent across concentrations. The mean percentage ratios of *R-*PZQ in blood or DBS compared to plasma, display ratios of 79.0 and 89.6%, respectively (Table 3). There is therefore a tendency for plasma samples to have slightly higher concentrations of *R*-PZQ than blood or DBS. For *S-*PZQ, the same pattern is observed, with slightly higher ratios than *R*-PZQ: 93.9 and 92.1% percentage ratios for blood and DBS, respectively. The metabolite *R-trans*-4-OH displays on the contrary higher ratios of blood or DBS to plasma of 122.0 and 110.6%, respectively. However, the 95% limits of agreement (LoA) all include 100%, except for R-PZQ in the blood *versus* plasma comparison (LoA = 59–99%). The LoA intervals are large and range up to 55–133% for the parent enantiomers and 94–145% for the metabolite in the blood-plasma ratios. LoA are slightly larger for DBS-plasma ratios.

**Fig 4:**
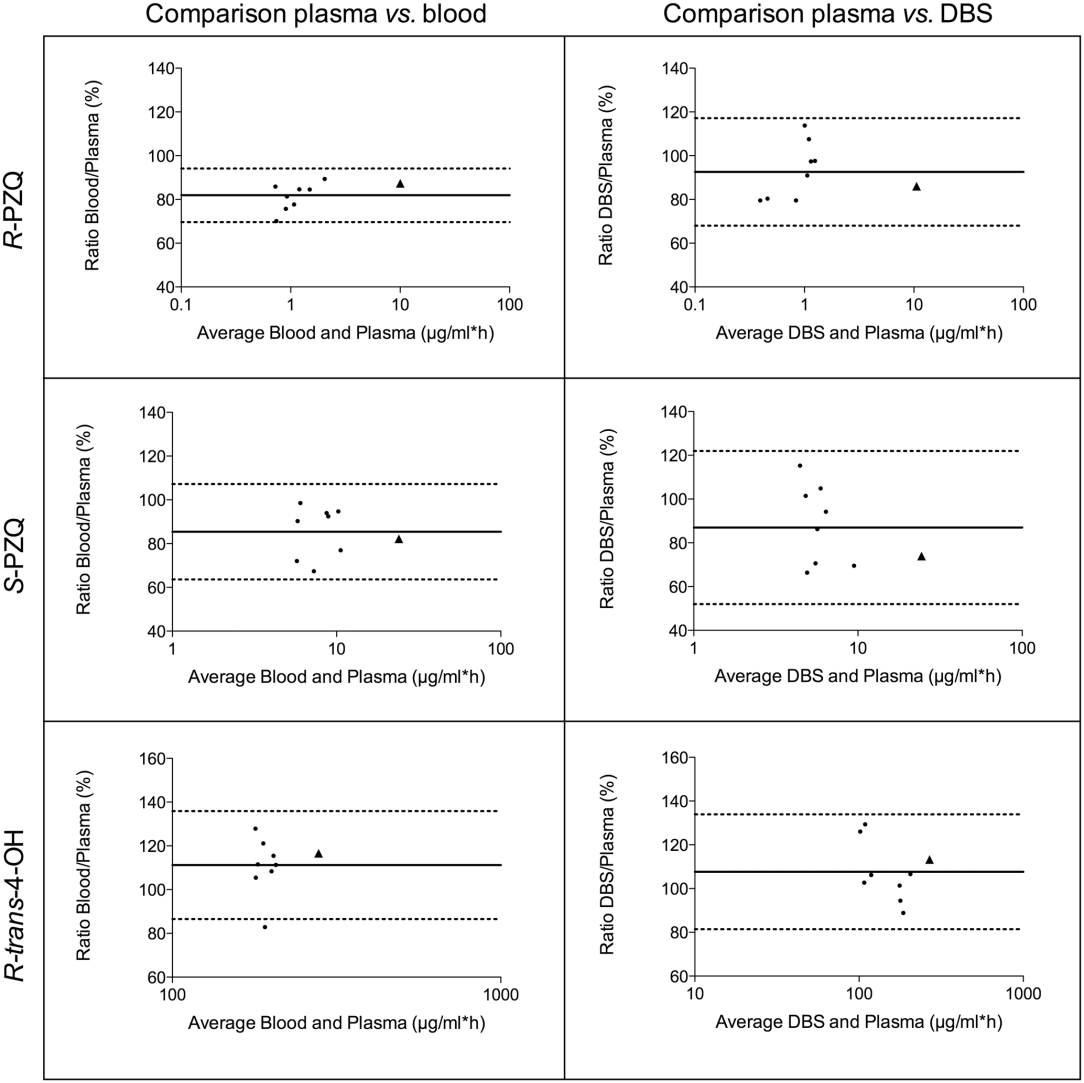
Bland-Altman plots of AUC values obtained from blood or DBS compared to plasma AUCs for *R*-PZQ, *S*-PZQ and *R-trans*-4-OH with mean ratio sketched with a solid line and 95% limits of agreement with dashed lines. Patient 2 is depicted with a triangle (▲).

Bland-Altman plots of AUC percentage ratios between plasma and blood or DBS are consistent across AUC values (Fig 4). The percentage ratios of blood or DBS to plasma for each PK parameter exhibit values between 80 and 120%, except in t_1/2_ DBS to plasma ratios for *R*-PZQ (122%) and *R-trans*-4-OH (75%, Tables 4 and 5). As for drug concentrations, the mean ratios of DBS or blood *versus* plasma tend to be lower than 100% for the parent enantiomers but higher than 100% for the metabolite. Only the t_1/2_ DBS to plasma ratios do not precisely follow this pattern, probably because they are calculated with 5 samples per patient instead of 10, driving therefore a higher estimation error. The LoA of the AUC ratios lie between 64–107% for the parent enantiomers and 87–136% for the metabolite in the blood-plasma ratios with a slightly wider range for DBS-plasma ratios. As for drug concentration results, all the LoA include 100%, except for *R-*PZQ in the blood to plasma ratio.

**Table 4:** Mean percentage ratios of AUC_0–24h_ values in blood or DBS compared to plasma and lower and upper 95% limits of agreement (LoA) with their respective 95% confidence intervals (CI).

	Mean ratio^a^ [%] (CI)		Lower LoA^b^ [%] (CI)		Upper LoA [%] (CI)	
Analyte	blood	DBS	blood	DBS	blood	DBS
*R*-PZQ	81.9 (76.8;86.6)	92.5 (82.9;102.2)	69.6 (61.4;77.9)	68.0 (51.3;84.7)	94.1 (85.8;108.9)	117.1 (100.4;133.8)
*S*-PZQ	85.4 (76.8;93.9)	87.0 (73.2;100.7)	63.6 (48.8;78.4)	52.0 (28.2;75.7)	107.2 (92.4;122.0)	122.0 (98.2;145.7)
*R-trans*-4-OH	111.2 (101.5;120.9)	107.6 (97.9;117.4)	86.5 (69.8;103.3)	81.4 (63.6;99.2)	135.9 (119.1;152.6)	133.9 (116.1;151.7)

^a^The percentage ratio is computed as blood or DBS AUC_0–24h_ values divided by plasma AUC_0–24h_ values averaged across patients and presented as percentage to the plasma values.

^b^The 95% limits of agreement for AUC_0–24h_ values were calculated with the conventional Bland-Altman method.

**Table 5:** Percentage ratios of t_1/2_, C_max_ and T_max_ in blood or DBS compared to plasma.

	t_1/2_		C_max_		T_max_	
Analyte	blood [%]	DBS [%]	blood [%]	DBS [%]	blood [%]	DBS [%]
*R*-PZQ	89.4	121.6	89.4	92.0	100.0	101.0
*S*-PZQ	81.8	100.0	81.8	96.5	100.0	99.4
*R-trans*-4-OH	105.2	75.3	105.2	103.5	115.4	111.5

### *In vitro* assessment of blood/plasma partition of PZQ

Blood to plasma ratios were consistent across both concentrations measured. *R-*PZQ displayed ratios of 83.1 ± 3.6 and 77.7 ± 3.9% for 0.05 and 0.5 μg/ml, respectively. The partition of S-PZQ in plasma was similar to *R*-PZQ, with ratios of 81.3 ± 5.4 and 74.5 ± 1.5% for low and high concentrations, respectively. The metabolite *R-trans*-4-OH showed a partition in plasma higher than the parent enantiomers, with values of 92.0 ± 6.0 and 87.5 ± 6.2% for 0.5 and 5 μg/ml, respectively.

## Discussion

PZQ is the only drug available for the treatment of opisthorchiasis, yet surprisingly preclinical and clinical work including PK studies are sparse. We conducted for the first time a PK study in patients infected with *O*. *viverrini* treated with three doses of PZQ and studied the enantioselective drug disposition in blood, plasma and DBS.

The only other PK study conducted with *O*. *viverrini*-infected patients so far examined the kinetic disposition of the racemic drug after a single oral dose of 40 mg/kg [20]. Patients were of similar age and weight as in our study, but with a higher proportion of males. The authors observed a C_max_ for racemic PZQ of 0.9 and 1.1 μg/ml in early (asymptomatic) and acute (moderately advanced) infection, respectively which does not differ from the value observed in our study, 1.1 μg/ml for *R*-and *S-*PZQ combined. A half-life value of 2.3 and 3.8 h previously observed for the racemic parent compound in early and acute infection [20] is as well consistent with a half live of S-PZQ of 3.3 h (R-PZQ is eliminated much faster, due to different enzymes kinetics than *S*-PZQ) in our study. Given that a dose of 40 mg/kg corresponds to 53% of the dose administered in our trial, the AUC_0–24h_ range observed in our study (6.1–26.3 μg/ml) is closer to that previously reported for patients with acute opisthorchiasis (2.5–15.6 μg/ml) than that in patients with asymptomatic opisthorchiasis (1.6–5.0 μg/ml) [20]. This result is not surprising, given the disease prevalence in the region and the age of the patients, for which acute cases are expected to be frequent [5, 35, 36].

We observed a high variability in analyte concentrations between patients. This is often observed in PK studies with PZQ and is likely due to the high first-pass metabolism of PZQ in the liver or gut, with the activity of CYP 450 being highly dependent on the health, genetic and nutritional status of the patient [12]. Multiple dosing can also add to variability, since differences among patients in absorption and elimination as well as competition/saturation effects are common and exacerbate each other.

The high AUC_0–24h_ values of the PZQ enantiomers and metabolite determined for patient 2 (taking only two doses instead of three) might be explained by several factors. Firstly, this is the only patient suffering from a co-infection with whipworms and hookworms at follow up. Changes in drug metabolism due to immune reactions due to three co-existing parasites might be possible, as some immunomodulators were found to decrease hepatic activity [37, 38]. Secondly, patient 2 displays the highest weight to height ratio (body mass index of 35.2 kg/m^2^), which could lead to an overestimation of the effective drug dose, as it is often the case in overweighed patients [39]. Finally, this patient might have developed liver and intra-hepatic bile duct pathologies, thereby altering drug metabolism, as observed in patients infected with another liver fluke, *Fasciola hepatica* [37]. Although measurement of liver enzyme parameters and the physical examination did not identify this patient as a symptomatic opisthorchiasis case, ongoing liver pathology can not be ruled out [20]. In fact, the detection of hepatic abnormalities due to opisthorchiasis, such as fibrosis or moderate hepatomegaly, is recommended to be performed via ultrasonography (not done in the present study), as liver enzymes do not seem to be a reliable indicator for the pathology of this disease [7, 40].

Not surprisingly, patient 2 suffered from adverse events during the treatment course, as high C_max_ levels are often correlated with adverse events [41]. It might be worth highlighting that this patient as well all other study participants were cured following PZQ treatment. The high efficacy noticed with a triple dose of PZQ is in accordance with previous studies [42]. The patient with the highest infection intensity (patient 7: 13,920 EPG at baseline) displayed parent and metabolite AUC_0–24h_ values similar to the other patients with moderate EPG values (between 1,000 and 10,000 EPG at baseline), hence infection intensity does not seem to correlate with PZQ disposition.

The most striking result observed in the disposition of PZQ is the high concentration of *R-trans*-4-OH, culminating at 13.9 μg/ml. For comparison, a study from Lima *et al*. [21] conducted in healthy volunteers treated with a single oral dose of 25 mg/kg PZQ displayed a 10x lower C_max_ of *R-trans*-4-OH. This finding, which might be explained with changes in metabolism due to the liver disease, raises the question of the role of the metabolite in the opisthorchicidal activity of PZQ. *In vitro* and *in vivo* studies should be conducted to assess the activities of *R*-and *S-*PZQ and *R-trans*-4-OH against *O*. *viverrini*.

The incurred sample reanalysis revealed a proportion higher than 2/3 of the samples falling into the acceptance criterion of deviating no more than 20%. These results demonstrate that the measurements are reliable and that there are no major problems in sample handling, processing or analysis. The hematocrit of 35% used for the calibration line and the 25–50% range of hematocrits used for the quality controls reflects values in our patients (mean hematocrit of 35.5 ± 4.1%), and more generally values encountered in Southeast Asia. For example, in Thailand, the mean hematocrit in men is between 42 and 47%, while in women it lies between 37 and 39% [43].

All the LoA intervals included 100%, indicating no difference between DBS or blood compared to plasma concentrations, except for R-PZQ in blood. The LoAs observed were wide, which can be explained by the additive measurement errors in each matrix. When validating a bioanalytical method, the accepted measurement variability is of ± 15%. This translates to indicative maximal LoA of 71–129% (calculated using the conventional Bland-Altman formula with SD = 15%), which is broadly similar to the results observed in this study. The wider LoA and confidence intervals for DBS-plasma compared to blood-plasma AUC_0–24h_ ratios reflect the half as small sample size for the estimation of DBS AUC_0–24h_s compared to blood AUC_0–24h_s samples. In light of these observations, we estimate that there is a general agreement between matrices and that DBS is a valid surrogate to venous sampling.

In the Bland-Altman comparisons of blood *versus* plasma or DBS *versus* plasma, *R*- and *S*-PZQ displayed drug concentrations and AUC_0–24h_ percentage ratios of around 80% and *R-trans*-4-OH ratios higher than 100%. The higher concentrations observed for *R*- and *S*-PZQ when quantified in plasma compared to blood or DBS might arise from a very high affinity of the drug for plasma proteins. PZQ is highly protein-bound (~80%) [12]. Hence, red blood cells might have a slight diluting effect on PZQ concentrations, depending on the blood hematocrit [26]. For example, tasquinimod, an anticancer drug characterized by a very high plasma binding (>98%), revealed a blood:plasma ratio of 66% [44, 45]. This phenomenon was also observed in a study comparing DBS and plasma sampling with piperacillin and tazobactam in infants with DBS:plasma ratios between 50 and 60% [46]. Therefore, our results for the parent enantiomers are in line with previous observations in drugs with high plasma affinity and displayed an agreement between plasma and blood or DBS of around 80–90%. In contrast, *R-trans*-4-OH did not follow such pattern and displayed higher concentrations in blood or DBS than in plasma, which likely indicates a lower affinity for plasma proteins than its parent molecule and a higher repartition in erythrocytes.

The *in vitro* evaluation of PZQ partition between plasma and erythrocytes highlighted a higher affinity of the enantiomers to plasma, which echoes the observations in patients described above. Considering that PZQ is bound to 80% to plasma proteins [12] and that acetonitrile precipitation extracts both the unbound and bound fractions, the blood to plasma ratios between 75 and 83% indicate that penetration of the free fraction into erythrocytes is very limited to almost absent. On the other hand, the metabolite *R-trans*-4-OH displays *in vitro* an almost even distribution in all blood compartments, while in patient samples this ratio is slightly more biased towards erythrocytes.

In conclusion, we have shown that DBS is a valid alternative to plasma sampling for PK studies with PZQ. Additional studies are warranted to estimate the kinetic disposition of patients after different PZQ dosing schemes and to investigate the PK/PD relationship, in particular the role of *R*–*trans*-4-OH in the opisthorchicidal activity of PZQ.
